# Canonical and Non-Canonical Roles of GRK2 in Lymphocytes

**DOI:** 10.3390/cells10020307

**Published:** 2021-02-03

**Authors:** Jing Cheng, Peter C. Lucas, Linda M. McAllister-Lucas

**Affiliations:** 1 Division of Hematology-Oncology, Department of Pediatrics, University of Pittsburgh School of Medicine, Pittsburgh, PA 15224, USA; linda.mcallister@chp.edu; 2 Divisions of Molecular Genomic Pathology and Experimental Pathology, Department of Pathology, University of Pittsburgh School of Medicine, Pittsburgh, PA 15224, USA; lucaspc@upmc.edu; 3 UPMC Hillman Cancer Center, Pittsburgh, PA 15232, USA

**Keywords:** Lymphocyte, GPCR, cell signaling, lymphoma

## Abstract

G protein-coupled receptor kinase 2 (GRK2) is emerging as a key integrative signaling node in a variety of biological processes ranging from cell growth and proliferation to migration and chemotaxis. As such, GRK2 is now implicated as playing a role in the molecular pathogenesis of a broad group of diseases including heart failure, cancer, depression, neurodegenerative disease, and others. In addition to its long-known canonical role in the phosphorylation and desensitization of G protein-coupled receptors (GPCRs), recent studies have shown that GRK2 also modulates a diverse array of other molecular processes via newly identified GRK2 kinase substrates and via a growing number of protein-protein interaction binding partners. GRK2 belongs to the 7-member GRK family. It is a multidomain protein containing a specific N-terminal region (referred to as αN), followed by a regulator of G protein signaling homology (RH) domain, an AGC (Protein kinase A, G, C serine/threonine kinase family) kinase domain, and a C-terminal pleckstrin homology (PH) domain. GPCRs mediate the activity of many regulators of the immune system such as chemokines and leukotrienes, and thus GRK proteins may play key roles in modulating the lymphocyte response to these factors. As one of the predominant GRK family members expressed in immune cells, GRK2′s canonical and noncanonical actions play an especially significant role in normal immune cell function as well as in the development and progression of disorders of the immune system. This review summarizes our current state of knowledge of the roles of GRK2 in lymphocytes. We highlight the diverse functions of GRK2 and discuss how ongoing investigation of GRK2 in lymphocytes may inform the development of new therapies for diseases associated with lymphocyte dysregulation.

## 1. Introduction

Ligand-bound G protein-coupled receptors (GPCRs) can activate various intracellular effector enzymes via coupling to heterotrimeric G proteins composed of α, β, and γ subunits. Specifically, the exchange of GDP for GTP on the Gα subunits leads to their dissociation from the βγ dimers, and the free Gα and βγ subunits then interact with and activate effector enzymes such as adenylyl cyclases, phospholipases, and others [1].

G protein-coupled receptor kinases (GRKs) promote the process of GPCR desensitization [2]. While agonist occupancy of GPCRs drives G protein signaling, it also triggers the translocation of GRKs, a family of serine-threonine kinases, from cytosol to plasma membrane where they phosphorylate the intracellular domains of activated receptors. GRKs and G proteins compete for interaction with the same site on the activated receptor [3]. Typically, GRK-mediated receptor phosphorylation uncouples the receptor from the G protein, which then terminates receptor signaling. In addition, receptor phosphorylation by GRKs facilitates subsequent binding of cytoplasmic β-arrestins which target GPCRs for clathrin-mediated endocytosis, a process that either leads to GPCR internalization and degradation or serves to dephosphorylate, re-sensitize, and recycle receptors back to the plasma membrane [4].

Seven GRK family members (GRK1–7) have been identified [5] and these proteins are grouped into three distinct subtypes. The GRK1 subtype includes GRK1 and GRK7, which are exclusively expressed in the visual system in retinal rods and cones, respectively [6]. The GRK2 subtype includes GRK2 and GRK3, both of which are ubiquitously expressed [7]. The GRK4 subtype includes GRK4, which is expressed primarily in the testis, cerebellum, and kidney [3], as well as GRK5 and GRK6, both of which are widely expressed. Beyond the canonical role in GPCR desensitization, emerging evidence suggests that GRK2, the most widely studied member of this family of kinases, also modulates multiple cellular responses in various physiological or pathological contexts by either phosphorylating non-receptor substrates or by directly interacting with signaling molecules. The overall impact of GRK2 on different cellular processes is the result of integration of its canonical and non-canonical functions and thus, it is important to understand how GRK2 acts in a stimulus, cell type, and context-specific manner. In this review, we discuss the canonical and noncanonical roles of GRK2 in lymphocytes. We also highlight a newly described role of GRK2 in influencing the pathogenesis of B-cell lymphoma.

## 2. Regulation of GRK2 Expression in Lymphocytes

GRK2 is expressed in many different tissues. The level of GRK2 is reported to be particularly high in cells of the immune system [8]. Various stimuli have been shown to regulate cellular GRK2 protein levels in lymphocytes. T lymphocytes express high levels of GRK2 and mitogenic stimulation of T cells induces a further increase in GRK2 protein. GRK2 protein levels in mononuclear leukocytes (MNL) [9] and peripheral blood lymphocytes (PBL) [10] are significantly increased after T cell mitogen, phytohemagglutinin (PHA) treatment and this occurs in association with increased synthesis of GRK2 mRNA. Stimulation of human T cells with anti-CD3 antibody and interleukin-2 has also been shown to up-regulate GRK2 mRNA and protein level and thereby result in increased GRK2 kinase activity [10], and GRK2 upregulation in T lymphocytes appears to be dependent upon activation of protein kinase C (PKC). In MNLs, short-term PKC activation (hours) increases the activity of GRK2, as measured by desensitization of the GPCR, β_2_ adrenergic receptor [11]. Intriguingly, chronic PKC activation with phorbol 12-myristate 13-acetate (PMA) and calcium ionophore decreases GRK2 protein level while increasing GRK2 mRNA expression [10]. These findings may indicate that posttranslational modification of GRK2 plays a role in reducing protein half-life, thereby providing negative feedback after chronic PKC stimulation [10]. These studies imply that the final impact of lymphocyte stimulation on GRK2 cellular level would result from combined effects on GRK2 gene transcription and GRK2 protein stability. The change in GRK2 activity observed upon T cell stimulation may indicate that GRK2 plays a key functional role in these cells. Activation of GRK2 in these cells can turn off certain regulatory stimuli, possibly through downregulation of specific GPCRs, which could interfere with T cell response to a specific antigen [9]. Alteration of GRK expression appears to be one mechanism through which T cells can adjust their response to specific stimuli.

In contrast to GRK2 upregulation after T-cell receptor (TCR) stimulation, a variety of other stimuli induce GRK2 downregulation in lymphocytes. For example, exposure of lymphocytes isolated from healthy donors to interferon-γ (IFN-γ) or interleukin-6 (IL-6) leads to reduced intracellular GRK2 protein levels [12]. Since the production of proinflammatory cytokines is increased in rheumatoid arthritis (RA), it has been proposed that the increase in cytokines including IFN-γ and IL-6 could drive the observed decrease in GRK2 expression and activity in MNLs isolated from RA patients [12]. Reactive oxygen species also play an important role in a variety of inflammatory conditions, including arthritis [13], and Lombardi et al. showed that oxidative stress, induced by exposure of lymphocytes to H_2_O_2_, results in the reduction of GRK2 protein level and activity and this occurs with no accompanying change in GRK2 mRNA expression [14]. Together, these findings suggest that lymphocyte GRK2 level can be reduced in response to inflammatory stimuli and this reduction can occur via mechanisms independent of GRK2 mRNA transcriptional downregulation.

Interestingly, multiple micro RNAs (miRNAs) have been implicated in downregulating GRK2 in lymphocytes and this may be important to the molecular pathogenesis of disorders associated with lymphocyte deregulation. For example, miR-125b, a miRNA implicated in the pathogenesis of B-cell malignancies [15], targets GRK2 for downregulation in a variety of leukemia cells, and in this context, GRK2 downregulation has been proposed to play a key role in inducing resistance to the chemotherapeutic agent, daunorubicin (DNR) [16]. Another miR-125 family member, miR-125a, also targets GRK2 in lymphocytes. High expression of miR-125a correlates with lower treatment response and shorter overall survival in acute leukemia, a correlation that may relate to downregulation of GRK2 by miR-125a [17]. Precisely how lowering GRK2 protein in lymphoid malignancy might promote drug resistance and shorter overall survival is not yet known.

Kaposi’s sarcoma-associated herpesvirus (KSHV) is an oncogenic herpesvirus that encodes multiple miRNAs that play key roles in viral pathogenesis. KSHV is associated with two B cell lymphoproliferative disorders known as primary effusion lymphoma (PEL) and multicentric Castleman’s disease (MCD). Interestingly, KSHV infection of B cells leads to decreased expression of GRK2 and concomitantly, increased levels of the GRK2-regulated GPCR, C-X-C motif chemokine receptor 2 (CXCR2). One of the over 25 miRNAs encoded by KSHV, miR-K12-3 (miR-K3), was specifically found to target GRK2 for downregulation, and this GRK2 downregulation and the resultant increase in CXCR2 signaling is required for KSHV viral latency. Because KSHV latency is critical for tumor development, these findings suggest that downregulation of GRK2 plays a key role in KSHV-driven B-cell lymphomas [18]. The identification of miRNAs that can control GRK2 dosage in lymphocytes is an active area of ongoing research and may have important implications for the role of GRK2 in B-cell malignancy.

Profound changes in GRK2 level and activity in immune cells have been described in several inflammatory diseases in both humans and animals. In these cases, downregulation of GRK2 in immune cells during inflammatory disease may represent an important adaptation mechanism for cells responding to a disease state, but at the same time, may also be maladaptive and contribute to disease progression [5]. Two prominent examples of inflammatory conditions in which GRK2 levels are seen to undergo downregulation include multiple sclerosis (MS) and rheumatoid arthritis (RA). GRK2 concentration is reduced in PBMCs of patients with active relapsing-remitting MS or with secondary progressive MS in comparison to healthy controls [19,20]. Similarly, the expression of GRK2 is significantly reduced in splenocytes from rats with experimental autoimmune encephalomyelitis (EAE), an animal model of MS [21]. The loss of GRK2 in GRK2^+/−^ mice induces earlier onset of EAE, though GRK2^+/−^ mice develop an acute phase of the disease without relapses [20]. Decreased GPCR kinase activity, accompanied by a decrease in GRK2 protein level, is similarly observed in PBMCs of patients with RA as compared to healthy controls [12]. In a rat model of adjuvant arthritis, GRK2 is also downregulated in splenocytes and mesenteric lymph node cells upon induction of arthritis [22]. These findings suggest that decreased GRK2 levels may play an important role in the development and/or progression of inflammatory disease. The fact that reduction in GRK2 protein level occurs after induction of arthritis in rats and returns to normal levels during disease remission suggests that inflammatory activity could directly alter GRK2 level in immune cells. In other words, the low GRK2 levels observed in lymphocytes are believed to be the consequence of the disease process and not a reflection of preexisting low levels of GRK2 in patients with arthritis [22]. Inflammatory activity during arthritis alters GRK2 level only in B cells and CD4^+^ T cells, the cell subsets that drive this disease, but not in CD8^+^ T cells or in thymocytes. Furthermore, no changes in GRK2 level/activity are observed in non-immune organs such as the heart and pituitary in these patients. These findings suggest that the inflammatory process induces a tissue and cell-specific down-regulation of GRK2 in the immune organs involved in the inflammatory response [22]. Of note, no changes of GRK2 mRNA levels were observed either in PBMCs of RA patients or in rat splenocytes during adjuvant arthritis, suggesting that the observed down-regulation of GRK2 associated with arthritis involves a process of posttranscriptional modification and/or protein degradation [12,22].

Our current understanding of the regulation of GRK2 levels within lymphocytes remains incomplete. GRK2 is known to be a short-lived protein with a half-life estimated at ~60 min in lymphocytes [23,24]. The half-life of GRK2 mRNA in lymphoid cells has been shown to be greater than 7 h [25]. Both recombinant GRK2 in HEK293 cells and endogenous GRK2 in Jurkat T cells are rapidly degraded by the proteasome [24]. With a relatively short protein half-life, post-translational modifications of GRK2 are potentially important factors in determining GRK2 protein level. In Jurkat T cells, the rate of GRK2 degradation is significantly increased in response to C-X-C motif chemokine 12 (CXCL12; also referred to as stromal-derived factor 1 (SDF-1α)) stimulation of C-X-C Motif Chemokine Receptor 4 (CXCR4) [26]. The precise mechanism by which this change occurs is not known. In contrast, treatment with tyrosine kinase inhibitor or inhibition of c-Src markedly reduces GRK2 degradation. Tyr 13, Tyr 86, and Tyr 92 have been identified as sites of phosphorylation of GRK2 by c-Src and mutation of these residues, to create non-phosphorylatable GRK2, results in significantly delayed GRK2 degradation after GPCR stimulation [26]. These results suggest that GRK2 phosphorylation by Src plays a critical role in regulating GRK2 degradation.

In summary, the above-described studies indicate that lymphocyte GRK2 is tightly regulated, both at the transcriptional and post-transcriptional level. Specific external stimuli have been shown to induce increased GRK2 level (e.g., TCR stimulation) whereas other stimuli induce a reduction of GRK2 level (e.g., IFN-γ or IL-6). Specific miRNAs have been identified that target GRK2 for downregulation in lymphocytes, and this mechanism of GRK2 regulation likely plays a key role in the pathogenesis of certain lymphoid malignancies. Further, certain inflammatory conditions, including rheumatoid arthritis and multiple sclerosis, are associated with downregulation of GRK2 in lymphocytes. Together, these findings strongly suggest that the level of GRK2 activity in lymphocytes plays a critical role in influencing lymphocyte function.

## 3. Canonical Roles of GRK2 in Lymphocytes

### 3.1. GRK2-Dependent Downregulation of Chemokine Receptors in Lymphocytes

GRK2 has been reported to control the intensity and duration of certain chemokine-triggered signaling events in lymphocytes during inflammation, acting most often in a canonical manner to trigger desensitization of chemokine receptors [27]. Down-regulation of GRK2 during chronic inflammation is thought to lead to increased or sustained activation of proinflammatory chemokine receptors [22]. Chemokines can also provide directional cues driving lymphocyte trafficking, migration into inflammatory sites, retention of hemopoietic precursors in the bone marrow, and anatomic compartmentalization of lymphocyte subpopulations in secondary lymphoid organs [28,29]. As such, excessive chemokine-dependent lymphocyte activation can contribute to inflammatory disease. Vroon et al. have shown that in comparison to wild-type T cells, the 50% decrease of GRK2 level in T cells from GRK2^+/−^ mice results in an increased chemotactic response of T cells to C-C chemokine receptor type 5 (CCR5) ligand, CCL4, and the CCR5/CCR1 ligands CCL3 and CCL5. Notably, the authors demonstrate that downstream signaling, specifically CCL4-induced calcium mobilization and activation of protein kinase B (AKT) and extracellular signal-regulated kinase (ERK) are significantly increased in GRK2^+/−^ T lymphocytes [30]. As noted above, it has been reported that patients with inflammatory autoimmune diseases, such as rheumatoid arthritis or multiple sclerosis, demonstrate a significant decrease in GRK2 level in their PBMCs as compared to healthy controls [12,19,20]. It is thought that reduction in lymphocyte GRK2 level during inflammation is associated with a pathologic increase in lymphocyte chemokine responses.

### 3.2. GRK2-Dependent Downregulation of S1PR1 in Lymphocytes

At least two types of GPCR ligands play essential roles in directing lymphocyte recirculation through lymphoid organs. The main signals that attract T cells into the lymph node are the chemokines CCL19 and CCL21, which bind to the GPCR, CCR7 [31] Conversely, sphingosine 1 phosphate (S1P), a lipid ligand for sphingosine 1 phosphate receptor-1 (S1PR1), promotes egress of lymphocytes from lymphoid organs into the vasculature where S1P is present at high levels in the blood [32,33]. S1P levels are maintained at a relatively high concentration in the blood plasma due to its production by endothelial cells and red blood cells, and its concentration drops by 100-fold in the peripheral lymph nodes as a consequence of high S1P lyase activity [34]. Chemokine and S1P gradients are essential for maintaining the proper distribution of lymphocytes between lymphoid tissue and blood.

**Figure 1:**
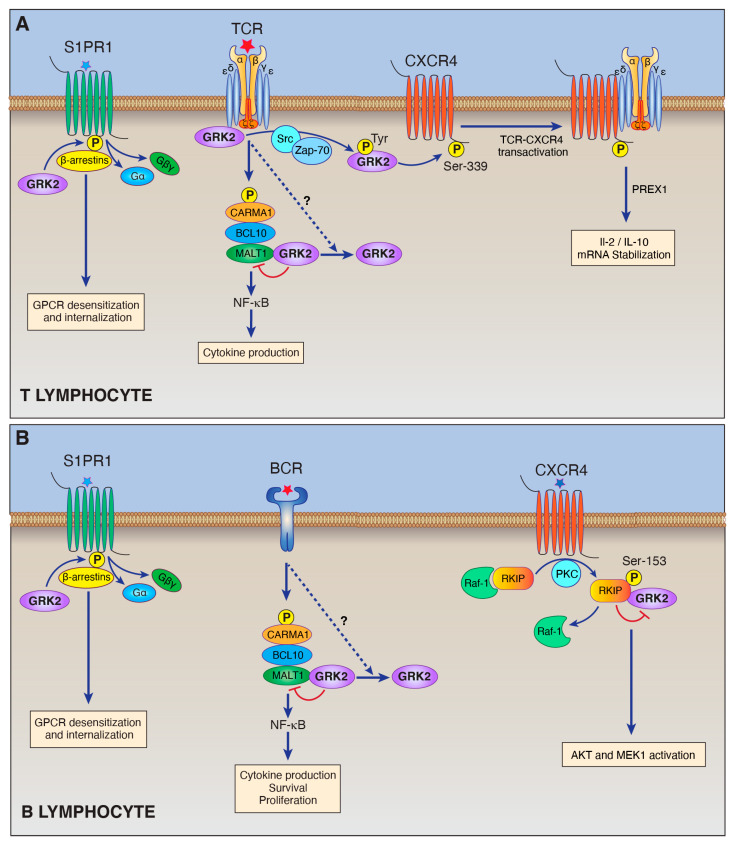
Schematic illustration of the roles of GRK2 in lymphocytes (see text for explanation of abbreviations). (**A**) GRK2 performs multiple functions in T lymphocytes. GRK2 carries out its canonical function of phosphorylating specific GPCRs including S1PR1. In this way, GRK2 works together with arrestins to trigger GPCR desensitization. GRK2 also has several non-canonical functions in T cells. GRK2 can directly associate with the TCR-CDε subunit. GRK2 also undergoes tyrosine phosphorylation in response to TCR stimulation, and phosphorylated GRK2 then phosphorylates and transactivates CXCR4, which drives TCR-CXCR4 complex formation and consequent downstream signaling. In addition, our laboratory recently demonstrated that GRK2 binds to MALT1, the effector molecule of the CARMA1-BCL1-MALT1 complex, and inhibits its activity. (**B**) GRK2 performs multiple functions in B lymphocytes. GRK2 phosphorylates specific GPCRs including S1PR1 to mediate GPCR desensitization. One of GRK2’s non-canonical roles in B-cells is its interaction with RKIP. PKC-dependent phosphorylation of RKIP results in RKIP dissociation from Raf-1 and association with GRK2. GRK2 binding to RKIP is thought to prevent GRK2’s binding and inhibition of MEK1 and AKT. In the B-cell malignancy, ABC-DLBCL, GRK2 may serve as a tumor suppressor by binding and inhibiting the MALT1 proto-oncoprotein.

In a landmark study, the laboratory of Jason Cyster demonstrated that GRK2 plays a critical role in promoting the return of lymphocytes from circulatory fluids back into lymphoid tissue, against the S1P gradient, by downregulating lymphocyte expression of S1PR1 on blood-exposed lymphocytes (Figure 1A,B) [35]. Specifically, the Cyster group demonstrated that deletion of GRK2 in lymphocytes results in reduced movement of T and B cells from blood into lymph nodes, and this movement is restored in S1P-deficient mice. Because germline GRK2 ablation results in embryonic lethality in mice [36], the Cyster lab conducted these studies by performing conditional deletion of GRK2 in T or B lymphocytes. They demonstrated that in circulating GRK2-deficient T cells, S1PR1 is resistant to desensitization, whereas wild-type T cells internalized the receptor within minutes upon exposure to high S1P levels in blood. In contrast, GRK2 deficiency did not affect many other chemokine receptors in blood T cells. Surface expression of CCR7 and CXCR4 on T cells from lymph nodes (LNs), sites of chemokine exposure, are similar between GRK2-deficient and control WT cells. Moreover, the ability of GRK2 deficient T cells to migrate toward sources of S1P in vitro was substantially increased compared to control, whereas migration toward CCR7 ligands was reduced and migration toward other chemokines remained unchanged. Accordingly, GRK2-deficient T cells showed reduced ability to enter lymph nodes upon intravascular transfer into wild-type mice [35]. Thus, the desensitization kinetics of S1PR1 allows T cells to dynamically shuttle between vasculature, with its high S1P concentration, and the lymphatic tissues, with its low S1P concentration, although the positional information imparted by the S1P concentration gradient between both compartments is static [37]. GRK2-mediated downregulation of S1PR1 expression allows T cells to ignore the high concentrations of S1P in the blood and to move into the lymph nodes, or in other words, GRK2-mediated S1PR1 phosphorylation and desensitization are required to prevent T cell persistence in the bloodstream.

Similar to the situation for T cells, GRK2-deficient B cells are also resistant to S1PR1 desensitization and demonstrate a reduced ability to enter lymph nodes. Interestingly, the Cyster group also showed that marginal zone B cell shuttling into follicles is disrupted by GRK2 deficiency or by mutation of a desensitization motif identified on the carboxy terminus of S1PR1. Building on these findings, the Kehrl laboratory further examined the impact of GRK2 on B cell trafficking using *Grk2^fl/fl^Mb-1-cre* mice. They found that GRK2 deficiency in developing murine B cells severely disrupts B cell trafficking and immune cell homeostasis and leads to a severe immune phenotype [38]. The mice demonstrated major reduction of bone marrow IgD^+^ cells, splenomegaly with severely disrupted architecture characterized by loss of white pulp and expanded red pulp, a deficit of Peyer patches, and abnormally small lymph nodes with significant reduction in B cell numbers [38]. Splenic B cells from these mice demonstrated reduction in S1PR1 internalization and enhanced migration to S1P. The loss of GRK2 led to a dramatic increase in the magnitude of S1PR1 signaling as assessed by pAKT, pERK, and intracellular Ca^2+^ levels. This enhanced signaling likely contributes to the hypermigratory responses of GRK2-deficient B cells to S1P [38].

Notably, in addition to promoting excessive S1PR1 signaling, the loss of GRK2 in B cells also led to impaired homeostatic chemokine receptor signaling. The expression of chemokine receptors CXCR4, CXCR5, and CCR7 on B cells from GRK2 deficient mice was found to be similar to controls [35,38]. However, the Kehrl laboratory found that the migratory response of GRK2-deficient B cells to multiple cytokines was suboptimal, with the specific migration to CXCL13 being most impaired [38]. Interestingly, blocking S1PR1 signaling with an S1PR1 antagonist partially corrected the poor response to CXCL13 in GRK2-deficient B-cells. The reversal of the CXCL13 migratory defect in GRK2-deficient B cells by an S1P antagonist suggests that GRK2-mediated S1PR1 desensitization somehow allows for normal chemokine receptor signaling [38]. In other words, in the absence of GRK2-mediated desensitization, the S1P/S1PR1 signal dominated over chemokine signaling [37]. Hopefully, future studies can elucidate the mechanism by which signaling through S1PR1 impairs (or shields) chemokine response. Overall, the unbalanced S1PR1 and homeostatic chemokine receptor signaling in the *Grk2^fl/fl^Mb-1-cre* mice causes a surprisingly severe B cell-trafficking defect that markedly disrupts normal immune organ architecture and function. Together, these studies clearly demonstrate that GRK2 plays a critical role in regulating the trafficking of both T and B cells.

## 4. Noncanonical Roles of GRK2 in Lymphocytes

In addition to its canonical role of regulating GPCRs via phosphorylation-dependent desensitization and internalization (see Figure 1), new roles for GRK2 in lymphocytes have also emerged. It is now apparent that GRKs possess GPCR-independent and phosphorylation-independent functions by engaging in a diverse repertoire of protein-protein interactions [7,39,40].

### 4.1. Association of GRK2 with the T Cell Receptor

Using co-immunoprecipitation and mass spectrometry, GRK2 was identified as binding to the CD3ε subunit of the T cell receptor (TCR) [41] (Figure 1A). The activation of T lymphocytes is a pivotal event in the immune response of all vertebrates to foreign and self-antigens. T cell activation is a multi-step phenomenon, triggered by effective contact of an external antigen with the TCR on the surface of a T lymphocyte. The predominant TCR isoform, the αβTCR, is a multimeric complex composed of the polymorphic α and β subunits and the CD3 γ, δ, ε, and ζ invariant chains. The extracellular peptide/major histocompatibility complexes (pMHC) interact with the α/β subunits of the TCR. While the TCRα/β heterodimer lacks inherent signal-transducing activity, the signal-transducing subunits CD3 γ, δ, ε, and ζ contain immunoreceptor tyrosine-based activation motifs (ITAMs) in their cytoplasmic domains. Following TCR engagement, tyrosine-phosphorylated ITAMs couple the TCR to downstream signaling machinery by binding key signaling molecules. In addition to ITAM, the CD3ε subunit possesses a basic rich stretch (BRS, also called a phospholipid-binding motif) and proline-rich sequences (PRS) in its cytoplasmic portion. DeFord-Watts et al. demonstrated that CD3ε constitutively associates with GRK2 in T lymphocytes through its membrane-proximal BRS motif [41]. The group proposes that this interaction of GRK2 with CD3ε may be a mechanism that somehow governs the cross-regulation of TCR and GPCR signaling in T lymphocytes.

### 4.2. GRK2-Dependent Regulation of theTCR-CXCR4 Complex

In T cells, stimulation of either CXCR4, the receptor for CXCL12/SDF-1α, or TCR results in the physical association of these two receptors and the activation of downstream signal transduction controlling cytokine production [42,43,44]. A recent report indicates that GRK2 plays a key role in mediating this process [43] (Figure 1A). TCR ligation results in the transactivation of CXCR4, defined as phosphorylation of CXCR4, that leads to the formation of TCR–CXCR4 complexes. This occurs in the absence of the CXCR4 ligand CXCL12/SDF-1α. The formation of these TCR–CXCR4 complexes activates a phosphatidylinositol 3,4,5-triphosphase (PIP2)-dependent Rac exchange factor (PREX1)/Rac-1 pathway and enhances cytokine mRNA stability [42]. Dinkel et al. proposed a model in which TCR-activated tyrosine kinases (Src and Zap-70) drive activation of GRK2′s kinase activity through its tyrosine phosphorylation [43]. GRK2 then transactivates CXCR4 by phosphorylation of CXCR4-Ser-339, which drives TCR-CXCR4 complex formation (Figure 1A). TCR-CXCR4 complexes induce PREX1 localization to the plasma membrane via a mechanism requiring PI3Kγ. Activation of the PREX1 signaling pathway downstream of the TCR-CXCR4 complex results in mRNA stabilization and thus robust IL-2 and IL-10 production [43]. siRNA-mediated depletion of GRK2 or treatment with the GRK2 kinase inhibitor, paroxetine, inhibits TCR-induced phosphorylation of CXCR4-Ser-339, TCR-CXCR4 complex formation, and cytokine production, suggesting that GRK2 kinase activity is required for both the TCR/CD3 signaling-induced transactivation of CXCR4 and for TCR–CXCR4 complex formation.

While the above signaling events emanating from TCR–CXCR4 complexes occur following TCR stimulation and in the absence of CXCR4 ligand, Kumar et al. have shown that TCR–CXCR4 complexes can also form in response to CXCR4 ligand activation, but with distinct downstream consequences [44]. Specifically, in response to stimulation with CXCL12/SDF-1α, CXCR4 closely associates with the TCR via a mechanism involving PI3K, and this colocalization permits CXCR4 to signal via ITAM domains of the TCR [44]. CXCL12 signaling through TCR leads to prolonged ERK activation and selectively mediates the transcription of Activator Protein 1 (AP-1) responsive gene products. Interestingly, TCR-CD3ε and CD3ζ subunits were shown to be in close association with CXCR4 after stimulation with CXCL12. While the work of Dinkel et al. demonstrates an important role for GRK2 in TCR ligation-induced CXCR4 transactivation and TCR-CXCR4 complex formation, the role of GRK2 in CXCL12 induced TCR-CXCR4 complex formation has not been investigated. In light of the prior discovery that GRK2 binds to CD3ε, it is tempting to speculate that this GRK2-CD3ε interaction could play a role in CXCL12-induced TCR-CXCR4 complex formation.

### 4.3. GRK2 Protein-Protein Interactions in Lymphoid Malignancy

Emerging evidence indicates that GRK2 acts as an onco-modulator, influencing multiple cellular functions related to the hallmarks of cancer, such as cell proliferation, cell survival, cell motility, cell metabolism, and angiogenesis, via its impact on cancer-relevant signaling networks [45,46]. Our laboratory recently identified the proto-oncoprotein, MALT1 (Mucosa-Associated Lymphoid Tissue Lymphoma Translocation 1), as a novel binding partner of GRK2 [47]. In normal lymphocytes, stimulation of the B or T cell antigen receptor (AgR) results in assembly and activation of the “CBM” intracellular signaling complex, which is composed of the scaffolding protein CARMA1, the adaptor protein BCL10, and the protease MALT1 [48,49]. CBM complex assembly and activation leads to downstream signaling including stimulation of the pro-survival NF-κB transcription factor, which subsequently results in lymphocyte activation and proliferation in response to antigen. MALT1 serves as the effector protein of the CBM complex, acting as a scaffold to recruit and activate downstream NF-κB signaling machinery [49]. In addition, MALT1 functions as a protease, whereby it enzymatically cleaves and inactivates multiple substrates, including several negative regulators of the NF-κB signaling pathway. Gain-of-function mutations that cause constitutive assembly of the CBM complex and deregulated activation of the MALT1 proto-oncoprotein underly the pathogenesis of a variety of lymphoid malignancies including activated B cell type-diffuse large B cell lymphoma (ABC-DLBCL) [50], MALT lymphoma [51], and mantle cell lymphoma [52].

Our findings indicate that the N-terminal region of GRK2, comprised of a short helix (αN) followed by RH domain, binds to the MALT1 death domain (DD) [47]. When bound to MALT1, GRK2 inhibits MALT1-dependent NF-κB activation. Notably, this N-terminal region of GRK2 is both necessary and sufficient for GRK2-mediated inhibition of MALT1 while the other domains within GRK2, such as the kinase and PH domains, are not required [47]. Interestingly, AgR stimulation, which promotes CBM complex assembly and MALT1 scaffolding and proteolytic activities, results in time-dependent dissociation of GRK2 and MALT1 in lymphocytes. These findings suggest that GRK2 binding to MALT1 inhibits CBM complex function, with the AgR-induced dissociation of GRK2 from MALT1 somehow allowing full activation of MALT1 and consequent downstream NF-κB signaling (Figure 1A,B). This is in contrast with the above-mentioned constitutive binding of GRK2 with the TCR CD3ε, which is not altered by antigen receptor stimulation [41]. While the impact of constitutive association of TCR CD3ε and GRK2 on downstream TCR-dependent signaling has not been investigated, our team found that GRK2 attenuates AgR-dependent CBM complex assembly, MALT1 scaffolding, and protease activity, NF-κB activation, and cytokine production in Jurkat T cells [47].

Further investigation unveiled a potential function of GRK2 as an onco-modulator/tumor suppressor by blocking MALT1 activity in MALT1-dependent lymphomas. First, we found that lower GRK2 mRNA expression is associated with significantly reduced survival in ABC-DLBCL [47]. Second, overexpression of GRK2 inhibits ABC-DLBCL cell proliferation while knockdown of GRK2 enhances ABC-DLBCL cell proliferation in vitro. Third, knockdown of GRK2 in ABC-DLBCL leads to enhanced tumor growth in vivo. It will be of great interest to elucidate the molecular mechanisms responsible for regulating GRK2 level within malignant lymphocytes as well as the precise mechanisms by which GRK2 binds and inhibits the MALT1 proto-oncoprotein.

In addition to this newly described role in MALT1-dependent lymphoma, protein-protein interactions involving GRK2 have also been implicated as influencing the pathogenesis of the related B-cell malignancy, chronic lymphocytic leukemia (CLL). In this context, the Raf-1 kinase inhibitory protein (RKIP) plays a key role as a binding partner and inhibitor of GRK2. This effect can be explained in a stepwise manner (Figure 1B): First, RKIP is highly expressed and constitutively phosphorylated on serine 153 by PKC in CLL cells [53]. Second, this phosphorylation results in a switch whereby RKIP releases its Raf-1 binding partner [54] in favor of binding to GRK2 [53]. Third, this RKIP binding to GRK2 blocks GRK2 kinase activity [53], thereby preventing GRK2 phosphorylation of GPCRs and stabilizing expression of receptors on the cell surface. RKIP binding to GRK2 also prevents GRK2 binding and inhibition of AKT and MEK1 signaling. A recent study by Crassini et al. showed that locostatin, an inhibitor of RKIP, downregulates phosphorylation of MAPK-ERK1/2 and AKT and induces apoptosis of chronic lymphocytic leukemia (CLL) cells [55]. The authors propose that locostatin may act by preventing RKIP binding to GRK2 and that loss of RKIP binding frees GRK2 to phosphorylate and downregulate GPCRs and to bind and inhibit AKT and MEK1 [55]. The authors further suggest that a detailed analysis of the sites of interaction between GRK2 and RKIP, MEK1, and AKT in CLL may inform new approaches to the treatment of CLL.

## 5. GRK2 Level in Lymphocytes as a Biomarker of Cardiovascular and Other Diseases

Lymphocyte GRK2 protein levels have been reported to mirror changes in GRK2 expression in other organs under several physiologic and pathologic conditions. As one example, myocardial GRK2 levels strongly correlate with GRK2 levels in peripheral blood lymphocytes, and GRK2 levels in peripheral blood lymphocytes are significantly higher in patients with myocardial infarction and heart failure (HF) [56]. Notably, GRK2 elevation in HF is associated with the loss of β-Adrenergic Receptor (β-AR) responsiveness and appears to increase with disease severity. Thus, lymphocytes may provide a surrogate biomarker for monitoring cardiac GRK2 in human HF [57,58,59,60]. As another example of lymphocyte GRK2 levels serving as a biomarker in cardiovascular disease, increased GRK2 expression in PBMCs has been reported in patients with hypertension [61]. In a large cohort of black American adults, lymphocyte GRK2 expression and activity directly correlated with systolic blood pressure [62]. This association was also observed in animal studies in which spontaneously hypertensive rats showed a significant increase in immunodetectable GRK2 in lymphocytes compared with normotensive rats [63].

Lymphocyte GRK2 levels may also serve as a biomarker for disorders other than cardiovascular disease. For example, GRK2 levels are higher in PBMCs from insulin-resistant patients as compared to healthy controls [64]. As another example, both GRK2 mRNA and protein expression are higher in lymphocytes from patients with Alzheimer’s disease (AD) compared to lymphocytes from healthy controls. Furthermore, lymphocyte GRK2 levels significantly correlate with the degree of cognitive decline in the AD patients [65]. Interestingly, a recent study has shown that acute mental stress significantly increased GRK2 levels in peripheral blood mononuclear cells (PBMCs) and had a positive correlation with epinephrine levels that also increased after the acute stressor. This study suggests a link between stress and cellular signaling that may be reflected by cellular GRK2 levels [66].

## 6. Conclusions

GRK2 performs a large and diverse set of roles, and the influence of GRK2 on cellular function is dependent on cell type, external stimuli and many other factors. Investigation of the many roles of GRK2 may inform the future development of new treatments for cardiovascular, neurologic, inflammatory, and malignant disease. In this review, we have presented a summary of our current knowledge of the role of GRK2 in lymphocytes. The body of work reviewed here indicates that GRK2 plays a complex and critical role in lymphocytes. Further, through its varied actions in lymphocytes, GRK2 influences the molecular pathogenesis of inflammatory and neoplastic disease. Despite recent advances in our understanding of GRK2 as a multifunctional signaling hub, many important questions remain the subject for future research. First, the mechanisms governing GRK2 level in lymphocytes, via transcriptional regulation and/or regulation of protein synthesis, activity, and stability, need to be unveiled. Investigation of miRNAs that regulate GRK2 expression is an active area of research and may have important implications for the influence of GRK2 in B-cell malignancy. Second, the mechanisms by which GRK2 controls the balance of signaling through S1PR and chemokine receptors in lymphocytes is not fully understood. Misbalanced S1PR1 and homeostatic chemokine receptor signaling in lymphocyte-specific GRK2 deficient mice causes a severe lymphocyte trafficking defect that disrupts normal immune organ architecture and function, suggesting that GRK2 plays a critical role in these processes. Lastly, precisely how GRK2 influences the functionality of its interaction partners in lymphocytes including TCR CD3ε, MALT1, RKIP, etc., are important questions whose answers will advance our understanding of normal immune response as well as the pathogenesis of lymphoid cancers. We have learned a great deal about GRK2′s varied roles in lymphocytes in the last decade and yet, much remains to be discovered.
